# Exercise inhibits tumor growth and central carbon metabolism in patient-derived xenograft models of colorectal cancer

**DOI:** 10.1186/s40170-018-0190-7

**Published:** 2018-11-15

**Authors:** Min Lu, Sydney M Sanderson, Amelia Zessin, Kathleen A Ashcraft, Lee W Jones, Mark W Dewhirst, Jason W Locasale, David S Hsu

**Affiliations:** 1 Division of Medical Oncology, Duke University Medical Center, 3008 Snyderman Building, 905 S. LaSalle St, Durham, NC 27710 USA; 2 Department of Pharmacology and Cancer Biology, Duke University, Durham, NC USA; 3 Center for Genomics and Computational Biology, Duke University, Durham, NC USA; 4 Department of Radiation Oncology, Duke University Medical Center, Durham, NC USA; 5 Department of Medicine, Memorial Sloan Kettering Cancer Center, New York, NY USA; 6 Weiil Cornell Medical College, New York, NY USA; 7 Levine Science Research Center, Room C270, 308 Research Dr, Durham, NC 27710 USA

**Keywords:** Colorectal cancer, Patient-derived xenograft, Exercise, Central carbon metabolism, Mitochondrial metabolism

## Abstract

**Background:**

While self-reported exercise is associated with a reduction in the risk of recurrence in colorectal cancer, the molecular mechanisms underpinning this relationship are unknown. Furthermore, the effect of exercise on intratumoral metabolic processes has not been investigated in detail in human cancers. In our current study, we generated six colorectal patient patient-derived xenografts (CRC PDXs) models and treated each PDX to voluntary wheel running (exercise) for 6–8 weeks or no exposure to the wheel (control). A comprehensive metabolomics analysis was then performed on the PDXs to identify exercise induced changes in the tumor that were associated with slower growth.

**Results:**

Tumor growth inhibition was observed in the voluntary wheel running group compared to the control group in three of the six models. A metabolomics analysis first revealed that central carbon metabolism was affected in each model irrespective of treatment. Interestingly, comparison of responsive and resistant models showed that levels of metabolites in nucleotide metabolism, known to be coupled to mitochondrial metabolism, were predictive of response. Furthermore, phosphocreatine levels which are linked to mitochondrial energy demands were associated with inhibition of tumor growth.

**Conclusion:**

Altogether, this study provides evidence that changes to tumor cell mitochondrial metabolism may underlie in part the benefits of exercise.

**Electronic supplementary material:**

The online version of this article (10.1186/s40170-018-0190-7) contains supplementary material, which is available to authorized users.

## Statement of translation relevance

In our current study, we used patient-derived xenograft model of colorectal cancer to model the effect of exercise on tumor biology. Coupled with metabolomics analysis of tumor samples, our current study provides the first characterization of the effect of exercise on the metabolic alterations in patient-derived xenografts.

## Background

Epidemiological studies have shown that exercise or physical activity can reduce both cancer incidence and progression in colorectal, breast, and prostate cancer [1, 2] and that the impact of exercise may differ as a function of tumor phenotype [3, 4]. There have been numerous molecular mechanisms proposed on the effects of structured exercise on tumorigenesis, and most of them have focused on systemic effects related to inflammation and alterations in circulating hormones such as insulin [5–7]. Although it is well appreciated that tumor cells alter their metabolism to support the demands of growth, survival, and proliferation [8, 9], whether exercise could affect cancer through alterations in metabolism remains unexplored in human cancer.

As is it well appreciated that tumor cells alter their metabolism to support the demands of growth, survival, and proliferation [8, 9], numerous studies have documented selective requirements of mitochondria for tumor growth [10, 11]. For example, metformin (an agent that targets the respiratory chain in the mitochondria and is thought in some instances to phenocopy the effects of exercise) has shown efficacy in chemopreventative settings in colorectal cancer patient populations suggesting that there may be clinical benefit from prescribed exercise [12]. Given that exercise alters systemic metabolism which should lead to concomitant changes in mitochondrial metabolism in all cells including cancer cells, a speculative hypothesis states that exercise may directly affect tumor cell proliferation by altering mitochondrial metabolism, but evidence to support such a theory is still lacking.

In our current study, we generated six colorectal cancer patient-derived xenograft (CRC PDX) models to determine the effects of exercise on tumor growth and intratumoral metabolomics alterations. Although the use of preclinical murine models to study effects of therapeutic intervention can be a powerful tool to identify and characterize potential therapies that may be clinically beneficial, the majority of xenograft models have been based on immortalized cancer cell lines engrafted in mice. Some of these cancer cell lines have been established for decades in cell culture, and thus may not fully capture the diversity of the disease and model an individual patient’s tumor [13]. In contrast, we and others have demonstrated that rapid engraftment of patient tumor samples into immunodeficient mice to develop PDXs may provide a more clinically applicable murine model to study potential therapeutic strategies [14–19]. Specifically, we have shown that many elements of the biology of PDXs are similar to that of the corresponding patient tumors, at both the histologic and molecular levels even after multiple passages [20, 21]. Using our established PDX approach coupled with metabolomics analysis of tumor samples, our current study provides the first characterization of the effect of exercise on PDX tumor growth as well as a global analysis of the metabolic alterations induced by exercise in both exercise-responsive and exercise-non-responsive tumors.

## Methods

### Collection of patient tumors

Tissues were collected from patients with histologically confirmed colorectal cancer (CRC) who had undergone complete surgical resections at the Duke University Medical Center between October 24, 2007 and June 9, 2011 under a Duke IRB protocol (Pro0002435). All participants provided written informed consent to participate in the study. Tumor tissues frozen in optimal cutting temperature (OCT) medium at the time of surgery were processed as (H&E) hematoxylin and eosin (Sigma) stained sections and histologically characterized by a board certified pathologist.

### Generation of patient-derived xenografts

All animal studies were performed at Duke University under an Institutional Animal Care and Use Committee (IACUC) approved protocol. Patient-derived xenografts (PDXs) of colorectal cancer explants were generated as described previously [20, 21]. Six surgically resected colorectal cancer samples (CRC240, CRC282, CRC361, CRC370, and BRPC12-146) were minced and injected subcutaneously into the flanks of 8-week-old NOD.CB17-PrkdcSCID-J mice (The Jackson Laboratory). Tumors were measured 2–3 times per week using a vernier caliper, and volumes were calculated using the formula, $V=\frac{\left({L}^2\times W\right)}{2}$ (*L* = longest diameter, *W* = shortest diameter)) until tumors reached ~ 1000 mm^3^. Tumors were then harvested and serially re-implanted until stable PDXs were established with a minimum of three generations. Harvested tumor tissues were frozen in optimal cutting temperature (OCT) medium and processed as hematoxylin and eosin (Sigma) stained sections and histologically characterized by a board certified pathologist.

### Physical activity studies

**Fig. 1:**
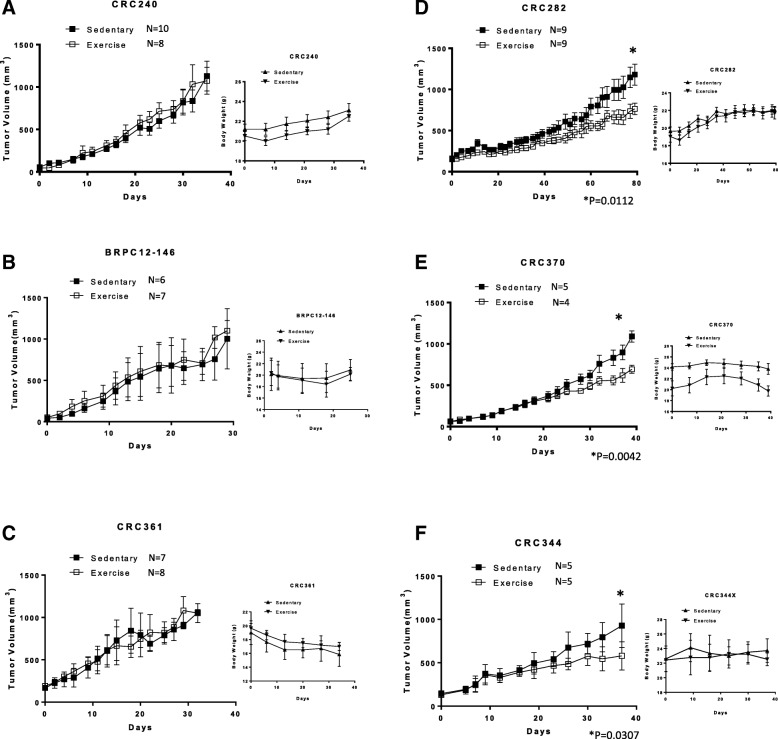
The effect of exercise on tumor growth in six different CRC PDX models. All CRC PDX were randomized into control and exercise treatment groups, with body weight and tumor size measured two or three times per week once treatment began. *N* = (number of mice used in this group) in each PDX models (**a**–**f**). Significance for tumor size difference at the endpoint between control and exercise groups in each PDX model was determined by unpaired Student’s *t* test (* indicates *p* < 0.05) (**a**–**c**) CRC240, BRPC12–146 and CRC361 had no response to exercise treatment (D-F), while CRC282, CRC370 and CRC344 had a significant response to exercise treatment with a decrease in tumor growth (all *p* < 0.05, unpaired Student’s *t* test)

Patient-derived xenografts (PDXs) of colorectal cancer used in the studies were developed by subcutaneous injection of homogeneous suspensions of previously developed PDXs and monitored until tumor formation began as described above. Mice in the control group were individually housed with an enrichment hut. Mice in the exercise group were singly housed with a wheel with a magnetic sensor to determine the distance each mouse traveled daily during the course of the experiment. Tumors were measured two or three times per week, and volumes were calculated by the equation $V=\frac{\left({L}^2\times W\right)}{2}$. For each independent PDX study, each group (control group or exercise group) contained 4–10 mice (Fig. 1). Endpoint of the study was determined using time to reach tumor volume of ~ 1000 mm^3^ or tumor ulceration. When tumors reached the endpoint, mice were euthanized under CO2 for 5 min. Tumors were harvested immediately (within 1 min after euthanization), snap frozen in liquid nitrogen, and stored at − 80 °C.

### Metabolite extraction

Tumor tissue (4 mg per tumor) was homogenized using a magnetized automatic homogenizer in 700 μL of ice-cold extraction solvent (80% methanol/water) and centrifuged for 10 min at 20,000 g at 4 °C. The resulting supernatants were transferred and split into two new Eppendorf tubes, and the solvent for each sample was evaporated in a Speed Vacuum. For polar metabolite analysis, the metabolite extracts were dissolved in 15 μL HPLC-grade water and 15 μL methanol/acetonitrile (1:1 *v*/*v*) (LC-MS optima grade, Thermo Scientific). Samples were then centrifuged for 10 min at 20,000 g at 4 °C and supernatants were transferred to liquid chromatography vials. The injection volume used for the polar metabolite analysis was 5 μL.

### Liquid chromatography

Ultimate 3000 HPLC (Dionex) with an Xbridge amide column (100 × 2.1 mm i.d., 3.5 μm; Waters) is coupled to Q Exactive-Mass spectrometer (QE-MS, Thermo Scientific) for metabolite separation and detection at room temperature. The mobile phase A reagent is composed of 20 mM ammonium acetate and 15 mM ammonium hydroxide in 3% acetonitrile in HPLC-grade water (pH 9.0), while the mobile phase B reagent is acetonitrile. All solvents are LC-MS grade, purchased from Fischer Scientific. The flow rate used was 0.15 ml/min from 0 to 10 min and 15–20 min, and 0.3 ml/min from 10.5–14.5 min. The linear gradient was as follows: 0 min 85% B; 1.5 min 85% B, 5.5 min 35% B; 10 min 35% B, 10.5 min 25% B, 14.5 min 35% B, 15 min 85% B, and 20 min 85% B.

### Mass spectrometry

The Q Exactive MS (Thermo Scientific) is outfitted with a heated electrospray ionization probe (HESI) with the following parameters: evaporation temperature, 120 °C; sheath gas, 30; auxiliary gas, 10; sweep gas, 3; spray voltage, 3.6 kV for positive mode and 2.5 kV for negative mode. Capillary temperature was set at 320 °C and S-lens was 55. A full scan range was set at 60 to 900 (m/z), with the resolution set to 70,000. The maximum injection time (max IT) was 200 ms. Automated gain control (AGC) was targeted at 3,000,000 ions.

### Metabolomics and data analysis

Data collected from LC-Q Exactive MS was processed using commercially available software Sieve 2.0 (Thermo Scientific). For targeted metabolite analysis, the method “peak alignment and frame extraction” was applied. An input file (“frame seed”) of theoretical m/z (width set at 10 ppm) and retention time of ~ 260 known metabolites was used for positive mode analysis, while a separate frame seed file of ~ 200 metabolites was used for negative mode analysis. To calculate the fold changes between different experimental groups, integrated peak intensities generated from the raw data were used. Pathway analysis was conducted using MetaboAnalyst software. Other quantitation and statistics were calculated using GraphPad Prism software. *T* test was used to compare metabolites intensity change between two groups (*p* < 0.05 was considered to have statistical significance).

## Results

### Characterization of six colorectal cancer PDX models

Six colorectal cancer (CRC) patient-derived xenograft (PDXs) were developed: CRC240, CRC282, CRC344, CRC361, CRC370, and BRPC12-146, as previously described [20, 21]. Tumor samples were derived from patients who underwent resection of their CRC liver metastasis, peritoneal implants, or primary CRC tumor. A summary of patient demographics is provided in Table 1. Similar to our previous work, pathological features between matched PDX and patient tumor were observed (Additional file 1: Figure S1A). We subsequently used these six CRC PDX models to study the effect of exercise on tumorigenesis.

**Table 1:** Demographics of patient-derived xenografts of CRC240, CRC282, CRC344, CRC361, CRC370 and BRPC12-146

	CRC240	CRC282	CRC370	CRC344	CRC361	BRPC12–146
Gender	Female	Male	Male	Male	Female	Male
Age	75	67	57	55	82	52
Race	Caucasian	Caucasian	Caucasian	Caucasian	Caucasian	Caucasian
Histology	Poorly differentiated adenocarcinoma	Well differentiated adenocarcinoma	Moderately differentiated adenocarcinoma	Poorly differentiated adenocarcinoma	Poorly differentiated adenocarcinoma	Poorly differentiated adenocarcinoma
Primary	Colon	Rectal	Colon	Colon	Colon	Colon
Metastatic site	Liver	Liver	Liver	Peritoneum	None	None
Microsatellite status	MSS	MSS	MSS	MSS	MSI	MSI

Control mice were singly housed with an enrichment hut, while mice assigned to the exercise group were singly housed with an exercise wheel and ran approximately 6 km per day (Additional file 1: Figure S1B, C). As the PDXs displayed different growth curves, tumor size of approximately 1000 mm^3^ was used as the primary endpoint. Three of the PDX models (CRC282, CRC344, and CRC370) were responsive to exercise as demonstrated by reduced tumor growth, while the remaining three models (CRC240, CRC361, and BRPC12-146) were non-responsive (Fig. 1). Based on these results, we categorized the mice into four subcategories: PDXs (CRC282, CRC344, and CRC370) with response to exercise (control—group 1 and exercise—group 2) and PDXs (CRC240, CRC361, and BRPC12-146) with no response to exercise (control—group 3 and exercise—group 4).

To identify metabolic alterations under exercise treatment, we performed liquid chromatography coupled with high-resolution mass spectrometry (LC-HRMS) in our 4 groups of PDX samples to profile the levels of 205 metabolites. We first performed an unsupervised hierarchical clustering on our samples which revealed widespread heterogeneity in metabolic programming between different PDX tumors with no distinct clusters distinguishing the four groups (Additional file 1: Figure S1B).

### Tumors from physically active mice exhibit globally altered metabolic profiles compared to tumors from control mice

**Fig. 2:**
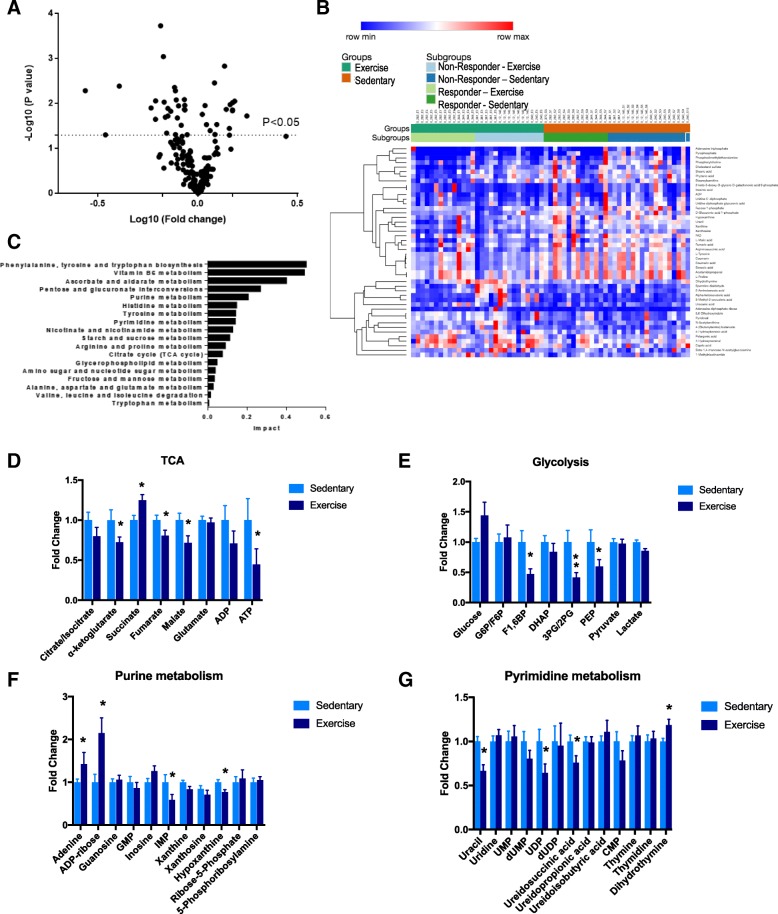
Tumors from exercised mice exhibit globally altered metabolic profiles compared to tumors from control mice**. a** Volcano plot comparing fold changes for metabolites between control and exercise groups across the six CRC PDX models. There were 47 significantly altered metabolites (*p* < 0.05, paired Student’s *t* test). **b** Heat map of significantly altered metabolites between exercise and control groups in the six CRC PDX models. The significantly altered metabolites are displayed using unsupervised hierarchal clustering. **c** Corresponding impacted pathways as determined by the list of 47 significantly altered metabolites. **d**–**g** Key metabolic pathways broken down by individual metabolites. Error bars are representative of standard error of mean (SEM); * indicates *p* < 0.05, paired Student’s *t* test

We next wanted to determine changes in metabolites between the exercise PDXs (groups 2 and 4) and control PDXs (groups 1 and 3). Supervised analysis revealed fold changes in metabolite levels revealed that 47 metabolites were significantly altered (*p* < 0.05, Student’s *t* test) by exercise (Fig. 2a). When these significantly altered metabolites were subject to unsupervised hierarchical clustering, a more defined pattern of metabolic alterations induced by exercise was observed (Fig. 2b). Pathway analysis revealed significant alterations in nucleotide (purine and pyrimidine), vitamin B6, and amino acid metabolism, as well as the TCA cycle (Fig. 2c).

### Exercise induces alterations to central carbon metabolism in responsive tumors

Based on these results, we examined representative metabolites of each significantly altered pathway to determine which metabolites were contributing to the observed shifts. Interestingly, the TCA cycle appeared to be downregulated by exercise, as the majority of the intermediates (with the exception of succinate and glutamate) were reduced (Fig. 2d). One of the most significantly impacted metabolites within this pathway was α-ketoglutarate, which is involved in numerous biological processes including acting as a cofactor for dioxygenase reactions and in the production of glutamate. We found a slight increase in glucose levels in PDX tumors extracted from exercised mice, while other glycolytic intermediates were either decreased or unchanged (Fig. 2e). Intratumoral pyruvate and lactate (the major byproducts of glycolysis) were not significantly elevated despite an increase in glucose uptake in the exercise group. It is possible that these intermediates were excreted from the tumor; alternatively, excess pyruvate and lactate can be converted into alternative energy sources such as acetyl-CoA (which feeds into fatty acid production), or into carbohydrates (such as glucose via gluconeogenesis) [22, 23]. Overall, these results indicate that exercise induces global alterations in intratumoral metabolic pathways involved in central carbon metabolism.

In addition to changes involved in central carbon metabolism, we found that exercise resulted in a small but significant decrease in tyrosine and proline, while levels of the other amino acids were not significantly impacted (Additional file 2: Figure S2A). Tyrosine is synthesized from phenylalanine and acts as a substrate for the production of catecholamines as well as the TCA intermediate fumarate, while proline can be synthesized from glutamate and has been shown to be increased in response to tissue repair [24]. Similarly, the acylcarnitines L-palmitoylcarnitine and stearoyl carnitine were found to be significantly reduced in tumors excised from exercised mice (Additional file 2: Figure S2B); acylcarnitines play an important role in providing substrates for fatty acid β-oxidation and have been explored as biomarkers for altered mitochondrial function [25], providing additional evidence for alterations in the TCA cycle and electron transport chain.

Finally, purine and pyrimidine metabolism (collectively referred to as nucleotide metabolism) were found to be some of the most impacted pathways. Looking at individual metabolites involved in purine metabolism, we found significant increases in adenine and the adenine-derivative ADP-ribose, while there were significant decreases in IMP and hypoxanthine (Fig. 2f). We primarily saw decreases in metabolites involved in pyrimidine metabolism, such as uracil, the uracil-derived molecule UDP, and ureidosuccinic acid (Fig. 2g). However, we also saw a significant increase in dihydrothymine, while thymine and thymidine were not significantly altered. While the functional significance of these data remains to be explored, these results indicate that exercise induces subtle but significant alterations in nucleotide precursor molecules, with generally opposite effects on purine- and pyrimidine-derived metabolites. Of note, the mitochondrion is an essential resource for nucleotide synthesis, providing further evidence for changes to mitochondrial metabolism in exercise-treated tumors.

### Tumors that are responsive to exercise demonstrate distinct metabolic profiles

**Fig. 3:**
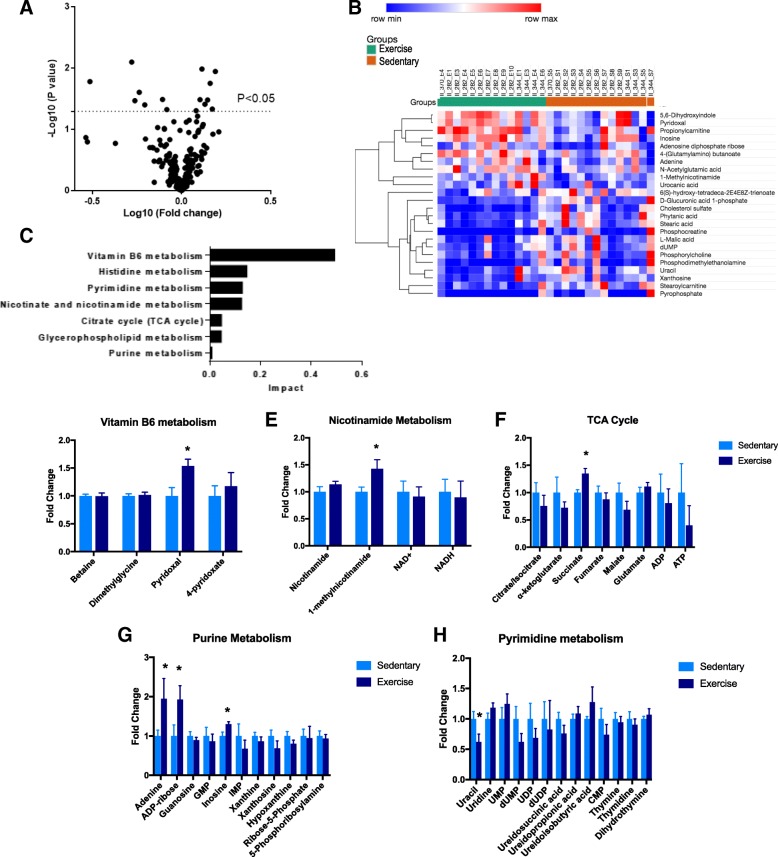
Exercise-responsive tumors demonstrate distinct metabolic differences. **a** Volcano plot showing fold changes in metabolites between control and exercise groups in the three exercise-responsive PDX models. Significantly altered metabolites (*p* < 0.05) determined using paired Student’s *t* test. **b** Heat map of integrated intensity values of the significantly altered metabolites between responsive control and exercise groups. **c** Corresponding impacted pathways as determined by the 15 significantly altered metabolites. **d**–**h** Key metabolic pathways broken down by individual metabolites. Error bars are representative of standard error of mean (SEM). * indicates *p* < 0.05, paired Student’s *t* test

Next, to examine the metabolic profiles of PDX tumors that were responsive to exercise (group 2) to PDXs that were not responsive to exercise (group 4), we analyzed the metabolomics profile of these two groups. Interestingly, we found a set of significantly altered metabolites (Fig. 3a, b) that primarily corresponded to a set of distinct metabolic pathways (Fig. 3c). Tumors excised from group 2 showed significantly increased levels of pyridoxal, a vitamin B6 molecule that plays a role in regulating intracellular homocysteine levels (Fig. 3d), and methylnicotinamide, a niacin derivative that has been shown to inhibit choline transport (Fig. 3e). Surprisingly, while the TCA cycle was found to be a highly affected pathway when comparing exercise to control groups of all six PDX models, analysis of the three exercise-responsive models (group 2) showed only a modest effect on the TCA cycle. The only significantly altered TCA intermediate was succinate (Fig. 3f); however, succinate serves as a proton donor to the electron transport chain, so alterations in intracellular levels can directly impact other critical energetic processes. We also observed similar patterns in nucleotide metabolism that were found in earlier analyses, such that adenine and ADP-ribose were elevated while uracil and dUMP were reduced (Fig. 3g, h), suggesting that intrinsic variation in nucleotide metabolism may track with responsiveness to exercise in the tumors.

**Fig. 4:**
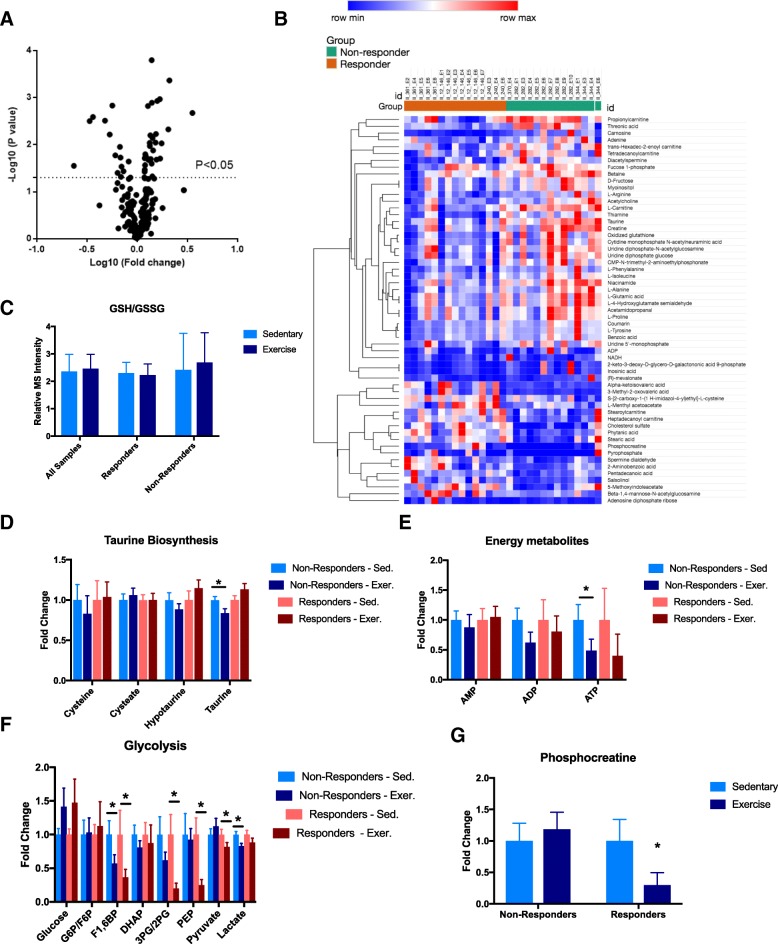
Exercise-responsive tumors exhibit differential metabolic responses to exercise compared to non-responsive tumors. **a** Volcano plot for fold changes of metabolites between exercise groups of the three responsive and three non-responsive physical activity PDX models. There were 24 significantly altered metabolites (*p* < 0.05, paired Student’s *t* test). **b** Heat map composed of fold changes (exercise versus control) of the 24 significantly altered metabolites between exercise-responsive and exercise non-responsive PDX models, displayed using unsupervised hierarchical clustering. **c** Oxidative stress as measured by ratio of reduced to oxidized glutathione levels. Error bars are standard error of the mean (SEM). **d**–**f** Key metabolic pathways broken down by individual metabolites, segregated into four experimental subgroups. Error bars are representative of standard error of mean (SEM). * indicates *p* < 0.05, paired Student’s *t* test. **g** Relative fold changes in phosphocreatine levels between control and exercise groups, compared between exercise-responsive and exercise-nonresponsive tumors. Error bars are representative of standard error of mean (SEM). * indicates *p* < 0.05, paired Student’s *t* test

Finally, we examined the differences in metabolic alterations induced by exercise between responsive (group 2) and non-responsive (group 4) PDX models and identified 58 metabolites that were differentially altered between the two groups (Fig. 4a, b). Many of these metabolites were involved in pathways that regulate energy and redox balance, such as cysteine metabolism, glycolysis, and fatty acid β-oxidation. Oxidative stress, as assessed by the ratio of reduced to oxidized glutathione, was unchanged by exercise in both exercise-responsive and exercise-non-responsive tumors (Fig. 4c). Closer examination of other cysteine-related metabolites showed that taurine and hypotaurine were slightly increased in exercise-responsive tumors (Fig. 4d), while they were reduced in their non-responsive counterparts; the significance of this differential alteration in cysteine metabolism is unclear, but could indicate that exercise-non-responsive tumors are able to use these downstream metabolites to help shuttle intracellular energy stores more efficiently.

ADP and ATP tended to be reduced in both exercise-responsive and exercise-non-responsive tumors (Fig. 4e). We also found that both exercise-responsive and exercise-non-responsive groups showed increased glucose levels, reflective of changes to mitochondrial metabolism (Fig. 4f). However, when we compared glycolytic intermediates, we observed that while both exercise-responsive and exercise-non-responsive tumors showed reductions in similar glycolytic intermediates, these reductions were more pronounced in the exercise-responsive tumors (Fig. 4f). Furthermore, phosphocreatine (an energy reserve important for rapid conversion of ADP to ATP under states of high energetic demand) was significantly reduced in the exercise-responsive group while essentially unchanged in the exercise-non-responsive group (Fig. 4f). While the functional implications of these alterations remain to be elucidated, together these preliminary data suggest that exercise-responsive tumors manifest through differential responses in energy metabolism with creatine metabolites possibly leading to biomarkers that may identify this response.

## Discussion

Most studies to date that have examined the effects of exercise on patient outcome have focused on reducing the risk or recurrence of tumor formation. For instance, one study found that having moderate to high levels of physical activity reduced the risk of developing colorectal cancer by age 30–40 years old, compared to those who led a more sedentary lifestyle [26, 27]. Similar findings have been shown for the breast, lung, and prostate cancers [28–32]. Furthermore, the majority of these studies have suggested that this beneficial effect is independent of body mass index (BMI) and overall physical fitness. However, little information is known about how exercise exerts these anti-tumor effects.

Previous studies using murine models of cancer to study the effects of exercise on intrinsic tumor biology have been reported. For example, it has been shown using a lung cancer cell line xenograft model that exercise activates p53 and increases apoptosis [33]. Another study found that in a breast cancer xenograft model, exercise appeared to reduce tumor growth by increasing microvessel density, vessel maturity, and perfusion thereby decreasing intratumoral hypoxia [34]. Interestingly, these findings were not found to be general, as some xenograft models did not display these effects while also displaying heterogeneous intratumoral changes in metabolism [35]. In our current study, we report the use of a patient-derived xenograft model system to study the effects of exercise on tumor growth, as well as the first evidence to show that certain intratumoral metabolic alterations are consistently induced by exercise independent of tumor growth in vivo*.*

Although we realize that our study was performed on a limited numbers of PDXs and that there are limitations in the steady-state metabolomics methods, our initial findings suggest that not all cancer patients may benefit from vigorous exercise, as only three of the six PDX models (specifically CRC240, BRPC12-146, and CRC361) showed significant reductions in tumor growth. Overall responsiveness to exercise therapy was heterogeneous across these models, and metabolism in each of these models in the presence and absence of exercise was markedly different, lending further appreciation to our understanding of the metabolic diversity in tumors. Our metabolomics analysis revealed that exercise induced changes in intratumoral central carbon metabolism in each model, independent of whether exercise was able to inhibit tumor growth. Nevertheless, such an effect on mitochondrial metabolism was not able to produce therapeutic results in all models, consistent with current thinking that the requirements of mitochondrial metabolism vary widely across patient populations. These limitations provide further insight into the challenges of considering exercise as a monotherapy and indicate the need for identification of potential biomarkers in patients that would benefit from this therapy.

Interestingly, metabolic features that were predictive of a response to exercise predominantly contained aspects of nucleotide metabolism. Previous studies have demonstrated that in many instances, nucleotide metabolism is limiting for cell proliferation and indeed targeting tumor metabolism with antimetabolites remain some of the most effective chemotherapies, including the frontline agent 5-fluorouracil used to treat advanced stage CRC. Since mitochondrial metabolism through several pathways (e.g., aspartate, serine, and glutamine metabolism) [36–41] is used to generate nucleotides, it is tempting to speculate that this link is differentially altered in the responsive tumors; additional flux analysis could validate this discovery.

Furthermore, we observed a significant decrease in phosphocreatine levels selectively in exercise-responsive tumors, possibly indicating altered energy metabolism. While the role of phosphocreatine is widely understood as an energy reserve in muscle, its role in cancer metabolism is unknown, likely due to the function of energy metabolism having been less appreciated relative to other roles for the mitochondria in cancer. While it is possible that these observed differences in energy metabolism are due to differential physiological host responsiveness to exercise independent of the inherent metabolism of the original PDX, each PDX group performed similar levels of exercise (Additional file 1: Figure S1B, C) and the metabolic consequences of exercise on whole-body physiology are reproducible and have been well-characterized [42], indicating that these alterations are likely tumor cell-autonomous. Therefore, the findings in this study suggest that energy metabolism could have a larger than previously appreciated role for the mitochondria in tumor biology.

In summary, these observations suggest that exercise appears to act on mitochondrial metabolism in PDX models of tumors and thus may in some instances have therapeutic properties through this mechanism. This may provide a therapeutic advantage for a subset of cancer patients, and could potentially be further enhanced when combined with other therapeutics or possibly diet. Future studies aimed at defining the mechanism of how exercise interacts with other therapeutic modalities would be of great clinical interest.

## Conclusions

Studies have shown that exercise plays an important role in inhibiting progression of colorectal cancer. Our current study shows that patient-derived xenografts (PDXs) can be used as a preclinical model to study the effects of exercise on tumor growth. Furthermore, coupling PDX studies with metabolic profiling provides a powerful platform to understand the effects of exercise on the metabolic activity of cancer. Future studies can now be designed to determine the effects of therapeutics based on metabolic profiling that can be enhanced with exercise in patients with CRC.

## Additional files

Additional file 1 Figure S1 The effect of exercise on tumor growth in six different CRC PDX models. (A) H&E slides of the six CRC PDX tumors. (B) Daily time spent in the light and dark. (C) Average distance run by three representative PDX models; each group ran approximately 5–8 km/day. (D) Heat map of integrated intensity values of 204 metabolites that were detected in tumors generated from six CRC PDX models, with metabolites grouped by unsupervised hierarchical clustering. (PDF 384 kb)

Additional file 2 Figure S2 Tumors from exercised mice exhibit globally altered metabolic profiles compared to tumors from control mice. (A) Fold changes of levels of individual amino acids (A) and acylcarnitines (B) between control and exercise groups. Error bars are representative of standard error of mean (SEM).(* indicates *p* < 0.05, paired Student’s *t* test). (PDF 676 kb)

## Abbreviations

CRC: Colorectal cancer
PDX: Patient-derived xenografts
