# Supplementary figures and images for: Exercise inhibits tumor growth and central carbon metabolism in patient-derived xenograft models of colorectal cancer
Source: Cancer Metab. 2018 Nov 15;6:14. doi: 10.1186/s40170-018-0190-7 (PMC6237036; doi:10.1186/s40170-018-0190-7)

Figure S1

A

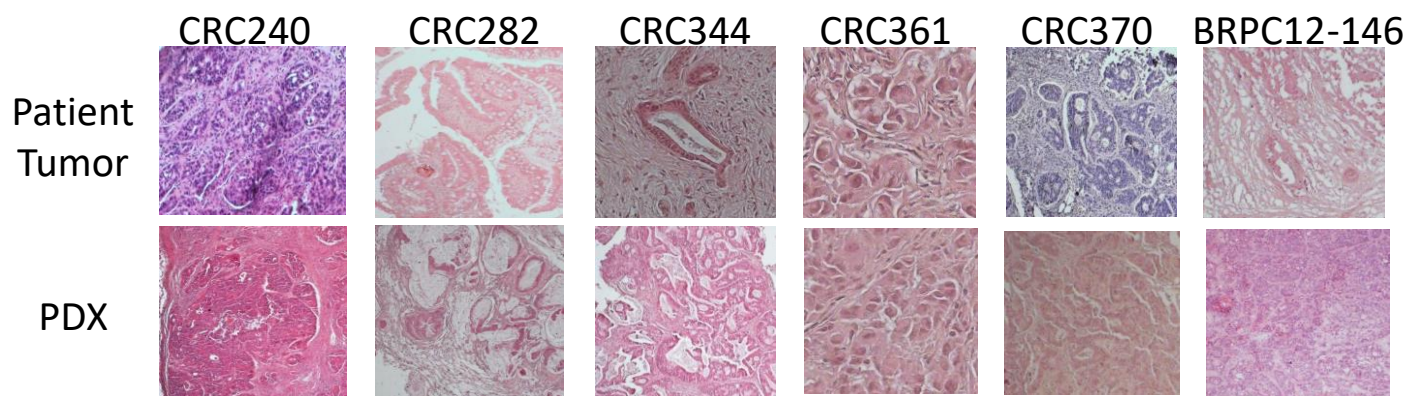

B

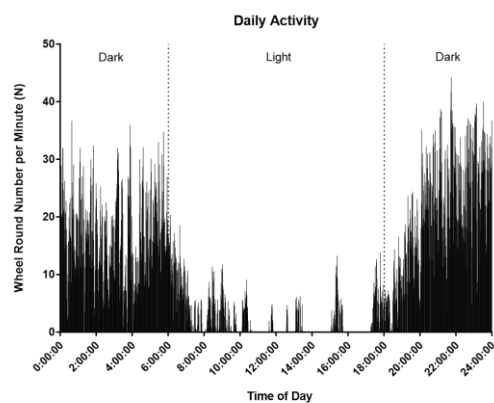

C

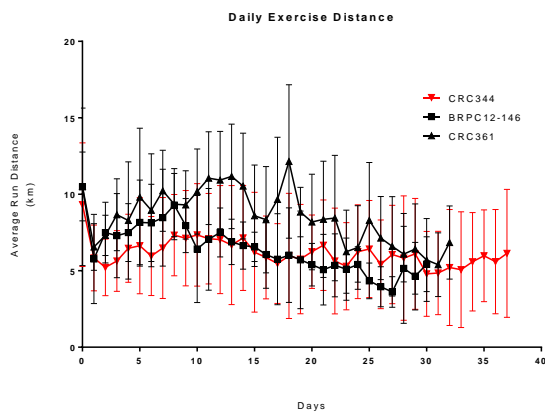

D

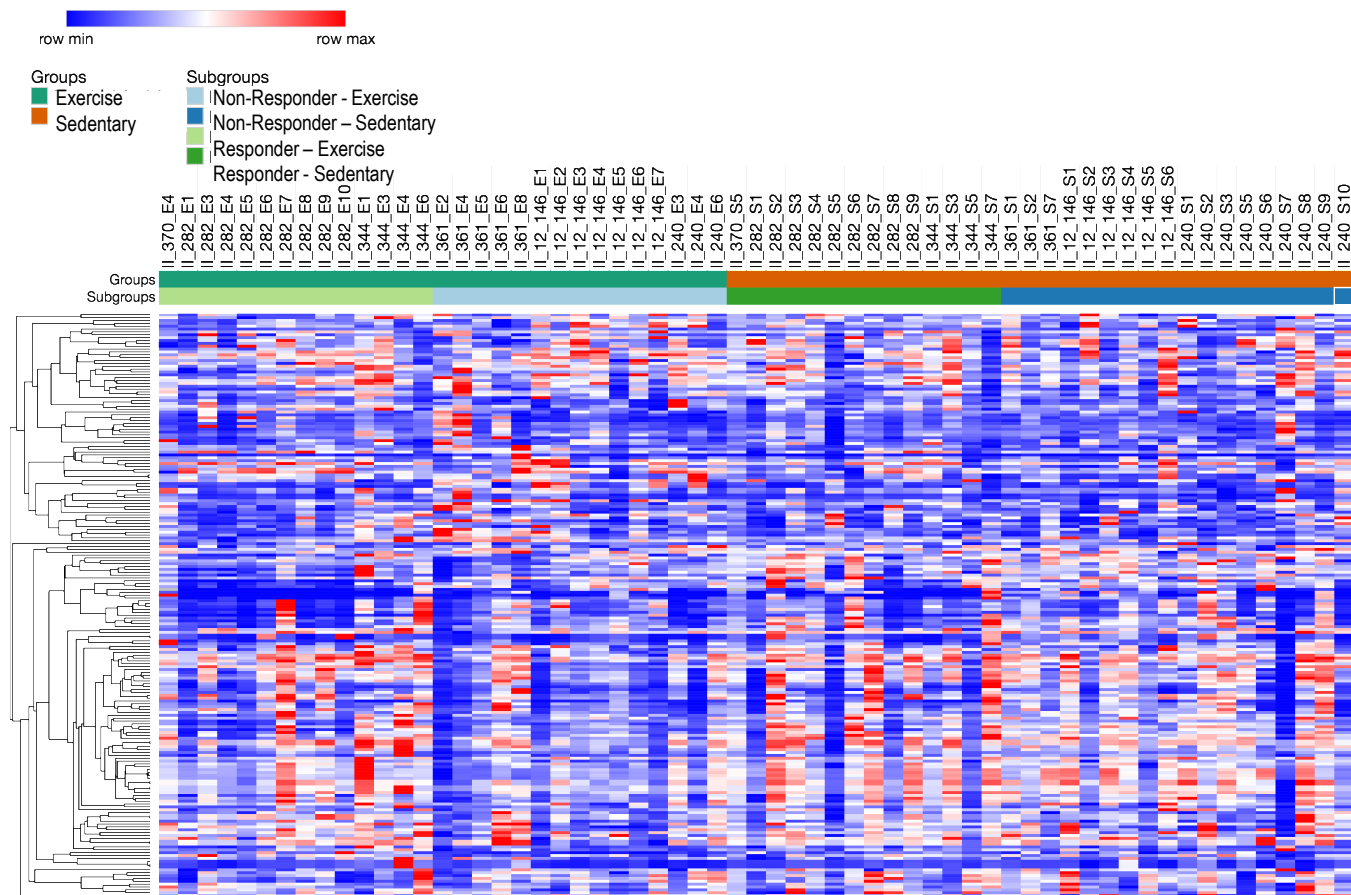

Supplement: Supplementary file 1 — Figure S1 The effect of exercise on tumor growth in six different CRC PDX models. (A) H&E slides of the six CRC PDX tumors. (B) Daily time spent in the light and dark. (C) Average distance run by three representative PDX models; each group ran approximately 5–8 km/day. (D) Heat map of integrated intensity values of 204 metabolites that were detected in tumors generated from six CRC PDX models, with metabolites grouped by unsupervised hierarchical clustering. (PDF 384 kb) [file 40170_2018_190_MOESM1_ESM.pdf]

Figure S2

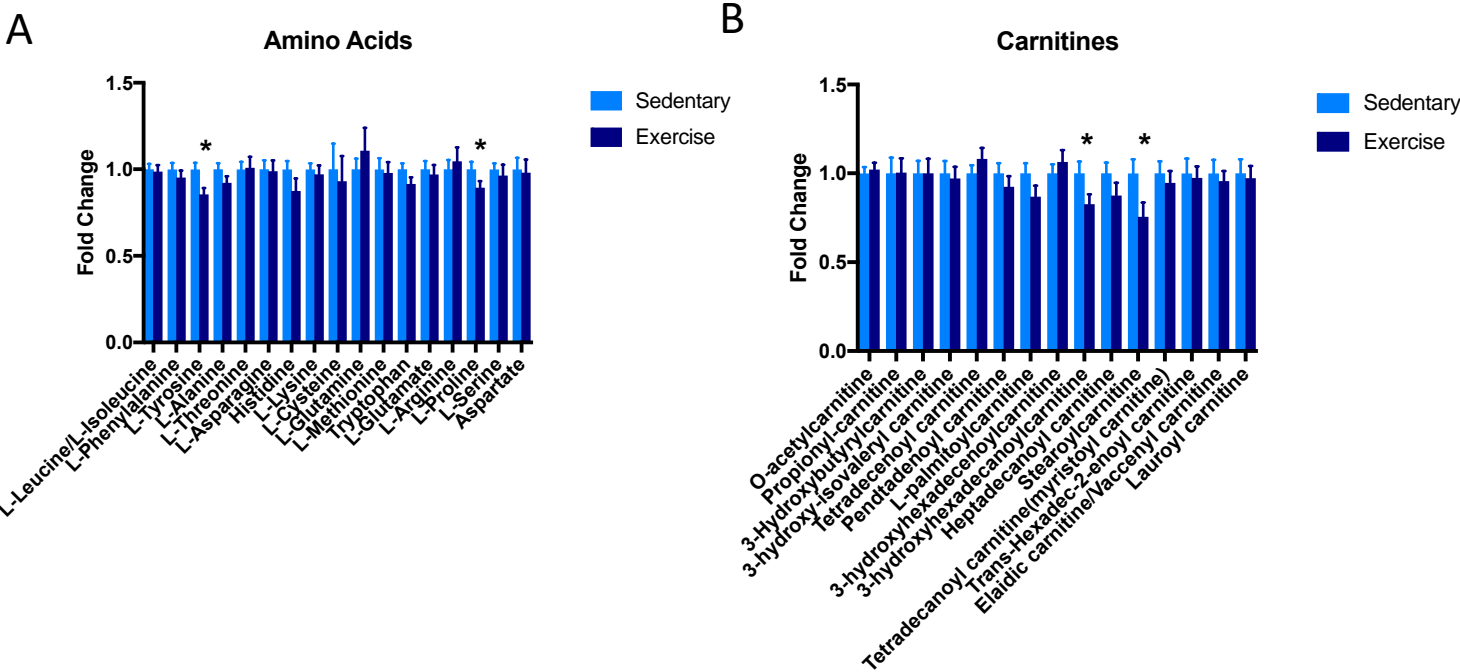

Supplement: Supplementary file 2 — Figure S2 Tumors from exercised mice exhibit globally altered metabolic profiles compared to tumors from control mice. (A) Fold changes of levels of individual amino acids (A) and acylcarnitines (B) between control and exercise groups. Error bars are representative of standard error of mean (SEM).(* indicates p < 0.05, paired Student’s t test). (PDF 676 kb) [file 40170_2018_190_MOESM2_ESM.pdf]
